# Infantile hemangioma: the common and enigmatic vascular tumor

**DOI:** 10.1172/JCI172836

**Published:** 2024-04-15

**Authors:** Annegret Holm, John B. Mulliken, Joyce Bischoff

**Affiliations:** 1 Vascular Biology Program and Department of Surgery, Boston Children’s Hospital, Boston, Massachusetts, USA.; 2 Department of Surgery, Harvard Medical School, Boston, Massachusetts, USA.; 3 Division of Pediatric Hematology and Oncology, Department of Pediatrics, University Hospital Freiburg, VASCERN-VASCA European Reference Center, Freiburg, Germany.; 4 Department of Plastic and Oral Surgery, Boston Children’s Hospital, Boston, Massachusetts, USA.

## Abstract

Infantile hemangioma (IH) is a benign vascular tumor that occurs in 5% of newborns. The tumor follows a life cycle of rapid proliferation in infancy, followed by slow involution in childhood. This unique life cycle has attracted the interest of basic and clinical scientists alike as a paradigm for vasculogenesis, angiogenesis, and vascular regression. Unanswered questions persist about the genetic and molecular drivers of the proliferating and involuting phases. The beta blocker propranolol usually accelerates regression of problematic IHs, yet its mechanism of action on vascular proliferation and differentiation is unclear. Some IHs fail to respond to beta blockers and regrow after discontinuation. Side effects occur and long-term sequelae of propranolol treatment are unknown. This poses clinical challenges and raises novel questions about the mechanisms of vascular overgrowth in IH.

## Infantile hemangioma

### Epidemiology.

Infantile hemangioma (IH) is the most common pediatric tumor. It affects approximately 5% of newborns, with a reported incidence range from 2% to 10%. IH is a benign vascular tumor, and for most children it poses no serious risk. About 10%–15% of IHs cause serious problems, such as cutaneous expansion, ulceration, and necrosis, particularly in facial features. Large tumors can cause high-output cardiac failure and consumptive hypothyroidism (1). The incidence of IH has increased over the last three decades (2). Major risk factors for IH include female sex, prematurity, low birth weight, European descent, multiple gestations, maternal progesterone therapy, and family history (3).

### Life cycle of IH.

**Figure 1:**
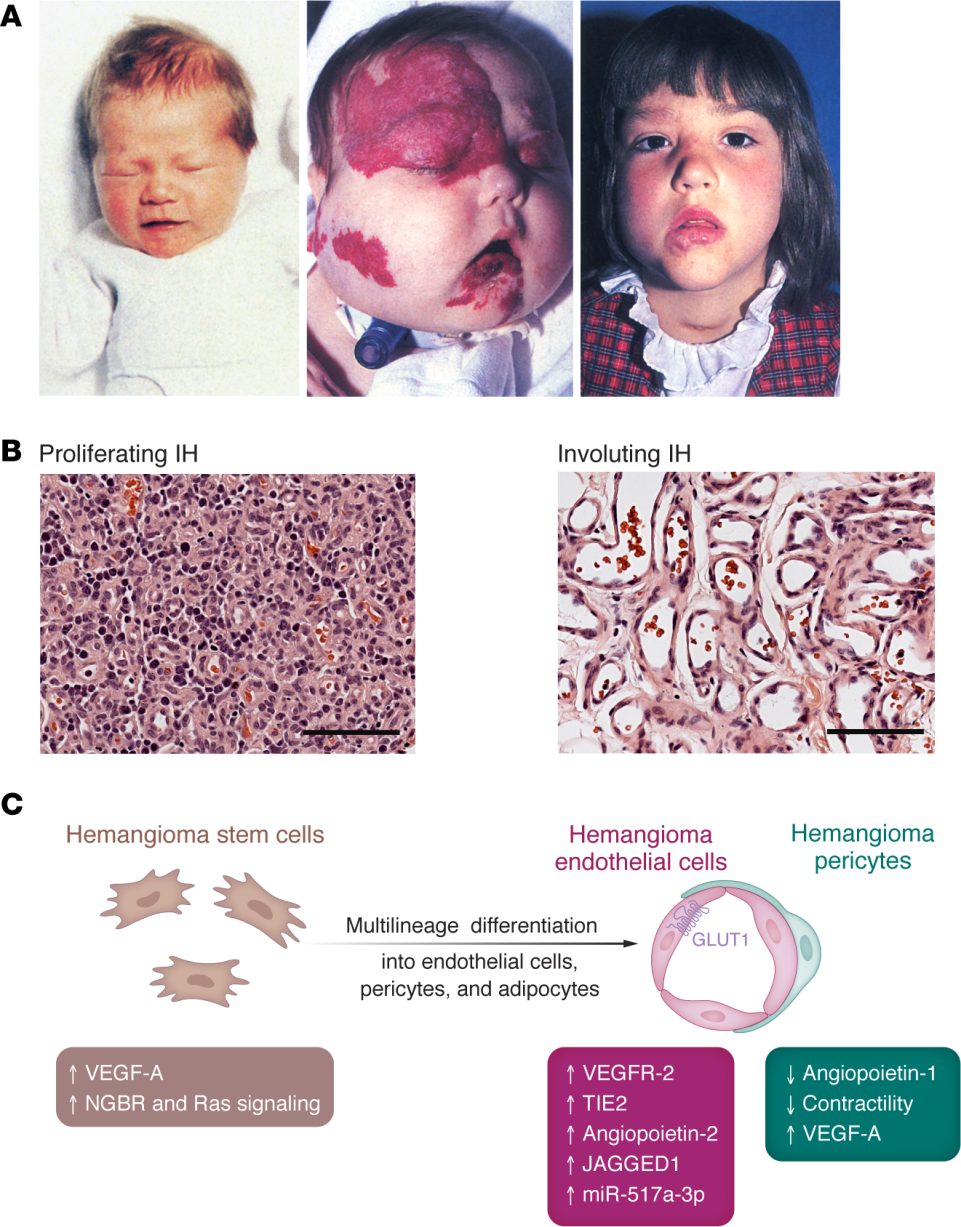
Life cycle, cellular components, and molecular players in IH. (**A**) Series of clinical images of a healthy female newborn (left) and the same individual at 5 months of age with an extensive facial and upper airway IH requiring tracheostomy (middle). This patient was treated with systemic corticosteroids, which was the mainstay of treatment at the time. The final image shows the patient at 5 years of age, with the IH in the involuted phase (right). Reproduced with permission from Elsevier (131). (**B**) H&E staining of proliferating and involuting IH demonstrates the differences in cellularity and vessel morphology in both phases in the IH cycle. Scale bars: 100 μm. Reproduced with permission from *Angiogenesis* (132). (**C**) Schematic of hemangioma stem cells, hemangioma endothelial cells, and hemangioma pericytes and molecular features of each cell type. Adapted with permission from the *British Journal of Dermatology* (133).

IH is usually not seen at birth. The tumor arises early in infancy (2–7 weeks of age), proliferates for 4–18 months of age, followed by slow involution over 3–9 years (4, 5), leaving behind a fibrofatty residuum in 50%–70% of cases, in addition to telangiectasia and redundant skin (5). Some children with involuted hemangioma require single or staged surgical resection (Figure 1).

Congenital hemangioma (CH) — a purpuric lesion, frequently with a circumferential halo — is an important differential diagnosis of IH (6). In contrast to IH, it arises in utero and is fully grown at birth; it typically decreases or is stable in size over time. CH can be complicated by ulceration, bleeding, congestive heart failure, and mild coagulopathy. Furthermore, CH is immune negative for the specific IH marker glucose transporter 1 (GLUT1) (7), and it may express somatic activating mutations in *GNAQ* and *GNA11*, which encode distinct α subunits of the heterotrimeric G protein Gα_q_ (8).

What prevents IH from growing in utero is not known, but some have speculated that postnatal hormonal changes might trigger tumor growth. In support of this, elevated levels of estrogen in the serum of infants with IH and increased estrogen and progesterone receptors in IH tissue have been detected (9). In vitro, estradiol was found to stimulate hemangioma endothelial cell (HemEC) proliferation (10) and hemangioma stem cells (HemSCs) to produce more VEGF-A and basic FGF and, in turn, to form more blood vessels when implanted in mice (11). These tantalizing observations and experimental results warrant further investigations.

### Classification.

IH is phenotypically classified according to depth and pattern of involvement. Superficial cutaneous IH is located in the papillary and reticular dermis, presenting as a red, finely lobulated plaque. Deep cutaneous IH infiltrates reticular dermis and subcutaneous tissue and can present as a skin-colored or bluish protruding mass (12). Patterns of involvement are focal, multifocal, or regional (13). The cutaneous distribution of IH seems to follow developmental units, including embryonic arteries, rather than specific dermatomes or Blaschko lines (14, 15). Large telangiectatic cutaneous IHs greater than 5 cm can be associated with malformations that are collectively known as PHACE(S) syndrome (posterior fossa anomalies, IH, head and neck arterial anomalies, cardiac anomalies, eye anomalies, sternal or supraumbilical defects). PHACE(S) syndrome affects 31%–58% of patients with large head or neck IH (16, 17). LUMBAR syndrome (lower body IH, urogenital anomalies/ulceration, myelopathy, bony deformities, anorectal malformations and arterial anomalies, renal anomalies) is the analogous association in the lumbosacral and perineural area. The IH presents with a segmental IH of the lumbosacral or perineal area, often associated with ulceration and an extension to one lower limb (18–20).

IH with minimal or arrested growth (IH-MAG) is a subtype that does not follow the typical life cycle of IH. IH-MAG can be mistaken for capillary malformations owing to the distinct flat telangiectatic appearance. It is present at birth, resolves spontaneously, and has a predilection for the lower body. The reason for the lower proliferative potential of IH-MAG is unclear; the distinct superficial anatomical distribution is suggested as one explanation (21, 22).

### Extracutaneous involvement.

A multifocal cutaneous distribution is seen in 10%–25% of infants with IH. The presence of 5 or more lesions is associated with internal organ involvement and is an indication for abdominal ultrasonography (23). The liver is most often affected, followed by the gastrointestinal tract, CNS (meninges), and mediastinum; the lung is rarely involved (24). Infantile hepatic hemangiomas (IHHs) are manifest in multifocal or diffuse patterns. They can be asymptomatic depending on size and distribution. Large IHHs, however, are at risk of life-threatening complications, including bleeding, high-cardiac output failure, hypothyroidism, and abdominal compartment syndrome (25). Less molecular information is available for IHHs because they are rarely resected.

### Complications.

IH can cause bleeding, ulceration, deformation, and obstruction with functional impairment. Extensive tumors, particularly intrahepatic, may result in cardiac overload.

Ulceration occurs most frequently during the early proliferating phase in both large and small IHs, especially in tumors located on the lower lip, neck, and anogenital region. Ulceration causes pain, bleeding, infection, and subsequent scarring (26). A mass effect can be critical depending on the location of the IH. Periocular IH can result in visual axis obstruction and corneal deformation leading to astigmatic amblyopia (27). IH located in the cervicomandibular region warrants particular caution for airway obstruction. The risk for airway IH is highest in patients with PHACE(S) syndrome (28). Consumptive hypothyroidism can occur in large regional IH and IHHs. The mechanism is overexpression of type 3 iodothyronine deiodinase, resulting in an increased deiodination of thyroxine and triiodothyronine (T3) to the biologically inactive reverse T3 and diiodothyronine (T2). Thyroid replacement therapy can be needed, along with treatment of the IH. Consumptive hypothyroidism typically resolves with tumor involution (29).

### Diagnosis.

Most IH are diagnosed clinically. In early stages, superficial IH, including IH-MAG, may be difficult to differentiate from a capillary malformation. Doppler ultrasound is particularly useful in the diagnosis of deep IH, revealing fast-flow characteristics, in part with arteriovenous shunts. This may lead to misdiagnosis of arteriovenous malformations (22). Extracutaneous IHs may require a more comprehensive diagnostic workup. A skin or organ biopsy can be performed if the diagnosis is in question to test for expression of GLUT1 in the tumor endothelium.

## Cellular studies of IH

Histologic study of IH in the proliferating phase reveals ongoing endothelial differentiation, with inchoate vessels expressing endothelial markers VEGFR2 and CD31 (30). In time, lumens enlarge, and the endothelium appears plump. Mural cells positive for α-smooth muscle actin (α-SMA), calponin, neural-glial antigen 2 (NG2), and PDGFR-β surround the abluminal side of the endothelium within a developing multilaminated basement membrane. Most vessels in the proliferating phase express GLUT1, a hallmark of hemangioma vessels (7), followed by a significant reduction in GLUT1^+^ vessels in the involuting phase (31). GLUT1 is a diagnostic tool to distinguish IH, but its role in the vascular overgrowth of IH has not been determined.

HemSCs, HemECs, and hemangioma pericytes (HemPericytes), as well as macrophages and telocytes have been isolated from excised IH specimens and characterized (Figure 1C). HemSCs expressing CD133, VEGFR2, CD90, and integrin α-6 recapitulate hemangiogenesis when implanted in immune-deficient nude mice, as shown by rapid formation of human GLUT1^+^ vessels and appearance of human adipocytes at 4–8 weeks (32–37). In vivo and in vitro experiments demonstrate the ability of HemSCs to differentiate into endothelial cells, pericytes, and adipocytes. In culture, HemSCs display a mesenchymal morphology and can expand from single cells to form clones. GLUT1^+^ endothelial cells isolated from proliferating phase IH revert to a HemSC mesenchymal morphology in culture, are clonogenic, and display the differentiative features of HemSCs (31). This indicates that the GLUT1^+^ endothelial cells lining hemangioma vessels retain stem cell properties while functioning as endothelium. Based on this finding, we designated the GLUT1^+^CD31^+^ cells in IH as *facultative stem cells* (31). This unique stemness concealed in GLUT1^+^ endothelium of IH may hold clues to the mechanisms of involution and the rebound phenomenon of IH.

HemECs proliferate and migrate robustly in vitro and express the endothelial markers CD31, VE-cadherin, and E selectin, as well as low levels of VEGFR1, which facilitates VEGFR2 signaling (38–40). When normalized to VEGFR2, IH tissues express relatively low levels of VEGFR1 compared with normal skin and placenta (41). In contrast to GLUT1-selected endothelial cells, HemECs purified without GLUT1 selection exhibit a stable endothelial cell phenotype but are unable to form perfused vessels in nude mice unless coimplanted with a mesenchymal support cell (42). In one study, HemECs resembled fetal ECs more than neonatal ECs, as shown by morphology and immunostaining (43). Proliferating phase HemPericytes express PDGFRβ, αSMA, calponin, and NG2 and reduced levels of angiopoietin-1 and exhibit decreased ability to induce endothelial quiescence and diminished contractility in vitro when compared with normal retinal or placental pericytes (44). In summary, HemECs and HemPericytes display increased angiogenic properties compared with normal skin ECs or retinal and placental pericytes. It is unclear to what extent these properties contribute to IH vessel formation.

Polarized M2 macrophages, identified as CD68^+^CD163^+^, are increased in proliferating versus involuting hemangiomas (45, 46). Further studies showed that the M2 macrophages promote endothelial differentiation of HemSCs. When mixed with HemSCs and coimplanted into mice, M2 macrophages increased microvessel density and delayed the appearance of adipocytes (46). These novel findings show an important paracrine role for M2 macrophages and suggest targeting M2 macrophages in the treatment of IH.

Lesser known telocytes have been observed in IH. These are mesenchymal cells characterized by long, thin processes that contact other interstitial cells (47). IH telocytes are described as perivascular and positive for aquaporin-1, CD34, PDGFR-α, and vimentin but not endothelial or macrophage markers. In vitro, IH telocytes interacted with endothelial cells and pericytes to form tubular structures. In the future, single-cell RNA-sequencing data sets from proliferating and involuting IH could provide a comprehensive view of the cellular composition of IH and how it changes during its regression.

## Molecular players in hemangioma

Hypoxia has been hypothesized to initiate hemangiogenesis, and the subsequent vascular growth has been viewed as an adaptive response to alleviate hypoxia (1, 48). Indeed, increased hypoxia-inducible factor 1α (HIF-1α) and HIF-2α have been detected in proliferating phase IH (49, 50). HIF transcriptional targets VEGF-A and GLUT1 have been detected as well. GLUT1 is expressed on CD31^+^ HemECs in vivo. In contrast, VEGF-A^+^ cells are distinct and found outside CD31^+^ vessels (51). This is corroborated in vitro, wherein VEGF-A mRNA and protein expression levels are high in HemSCs and low in HemECs (51). HIF transcription factors may activate genes to different extents depending on the cellular context, e.g., GLUT1 expression in newly differentiated HemECs and VEGF-A expression in HemSCs. shRNA knockdown of VEGF-A in HemSCs showed that VEGF-A is essential for hemangioma blood vessel formation in vivo in nude mice (51). Subsequent knockdown experiments showed that VEGF-mediated vasculogenesis requires VEGFR1 (52).

Hu and colleagues (37) discovered an integral role for the NOGOB receptor (NGBR) in coupling growth factor signaling to RAS activation in HemSCs. NGBR was shown previously to be required for association of prenylated RAS with the plasma membrane (53) and, hence, RAS activation. NGBR is strongly expressed in GLUT1^+^CD31^+^ vessels in proliferating IH. Knockdown experiments showed that NGBR is required for activation of RAS signaling in HemSCs and, in turn, for HemSC proliferation and migration. RAS signaling activates ERK1/2 and AKT, both of which are critical for normal vascular development and homeostasis but often go awry in vascular anomalies. Rescue with constitutively active HRAS or KRAS constructs confirmed the role of RAS signaling (37). Furthermore, siRNA knockdown of NGBR in HemSCs resulted in significantly fewer blood vessels when the cells were implanted in immune-deficient mice (37). These studies establish NGBR as critical integrator of growth factor/RAS signaling in HemSC differentiation and vasculogenic activities.

Juxtacrine signaling between endothelial cells expressing JAGGED1 and HemSCs expressing NOTCH3 induces HemSCs to differentiate into mural cells, with a phenotype similar to mural cells that surround IH vessels (36, 44, 54). Knockdown of NOTCH3 in HemSCs or a NOTCH3 decoy inhibitor showed that NOTCH3 in HemSCs is needed for mural cell differentiation and formation of IH vessels when HemSCs are implanted in immune-deficient mice (36). This novel study provided important insights into perivascular cell origins in IH. The renin angiotensin system has also been implicated in IH based on several findings. First, renin levels are high in infants less than 5 weeks of age, and levels tend to correlate with increased risk of IH. Downstream of renin, angiotensin II was shown to increase IH cell proliferation, providing an impetus for further investigation (55).

Noncoding RNAs have been implicated in hemangiogenesis. Strub and colleagues profiled miRNAs in 24 IH specimens versus normal skin adjacent to the tumor (56). They discovered that the C19MC miRNA mega cluster is overexpressed in IH but not in normal adjacent skin or in lymphatic malformation tissue. From the C19MC cluster, they showed that miR-517c-3p and miR-517a-3p are specifically expressed in IH tissue and not in seven other types of vascular anomalies. Furthermore, they showed that miR-517a/c-3p localized to GLUT1^+^CD31^+^ endothelial cells in IH specimens and in GLUT1^+^CD31^+^ cells isolated directly from IH tissue. Finally, as the C19MC miRNAs are found in the circulation, the authors measured miR-517-3p levels in plasma samples from ten infants with IH prior to the start of the study and after one and six months of oral propranolol therapy. Remarkably, plasma miR-517a-3p dropped significantly, concomitant with diminished IH. In one patient, IH regrowth occurred. In this case, miR-517a-3p was increased to levels more than double of what it was at the initiation of treatment. These remarkable findings suggest circulating C19MC miRNAs could be used to monitor IH growth, involution, potential for regrowth, and response to propranolol.

RNA binding proteins LIN28A and LIN28B are expressed in embryonic stem cells and known to interact and regulate the miRNA let-7. In turn, let-7 negatively regulates LIN28, forming a feedback loop to control stem cell self-renewal and differentiation. LIN28B was found highly expressed in proliferating phase IH tissue compared with normal skin, but in the same specimens, relatively low levels of let-7 were found (57). These investigators pursued experimental work in human induced pluripotent stem cells and showed that propranolol reduced LIN28B and increased let-7. This prompted them to speculate that propranolol therapy for IH may cause a similar decrease in LIN28 and increase in let-7 and, further, that LIN28B/let-7 balance may be critical for onset of the involuting phase (57).

## Evolution of therapy in IH

Pharmacologic treatment for IH has evolved steadily, aided by unexpected discoveries. By investigating the mechanism of action of serendipitously discovered drugs on the cellular constituents in IH, new molecular insights of this fascinating tumor have emerged.

### Corticosteroids.

Corticosteroids given orally or by intralesional injection were the mainstay of treatment for problematic IHs for about 5 decades, although they were never FDA approved for this indication. Corticosteroids were discovered serendipitously for IH by misdiagnosis, with the first patient reported in 1968 (58). In an effort to control thrombocytopenia (interpreted as immune thrombocytopenia) in a 13-year-old patient with a concomitant large “hemangioma,” corticosteroids were effective in both treating the thrombocytopenia and the vascular tumor (58). Based on these observations, corticosteroids were given to patients with problematic IH in the following years, independent of presence of thrombocytopenia (30).

Mechanistically, corticosteroids were shown to inhibit the expression of VEGF-A in HemSCs, and, in turn, VEGF-A was shown to be required for de novo HemSC vessel formation (51). The experimental results were as follows: treatment of HemSCs in vitro with dexamethasone dramatically reduced VEGF-A mRNA and protein; pretreatment of HemSCs in vitro with dexamethasone inhibited the ability of HemSCs to form blood vessels in vivo; and silencing VEGF-A in HemSCs inhibited blood vessel formation. Consistent with these findings, VEGF-A protein was detected in the proliferating phase but absent in involuting phase IH specimens. Treatment of problematic IHs with corticosteroids has been largely replaced by treatment with propranolol. Prednisone or methylprednisolone are still prescribed for patients with IH with contraindications or inadequate response to propranolol (59). Acute and long-term side effects of corticosteroids in infants, however, are to be considered, including hypertension, growth retardation, gastrointestinal irritability, respiratory distress, immunosuppression, and adrenocortical suppression (60). Administering both propranolol and corticosteroids has been tested. Results support giving the combination for particularly challenging and life-threatening tumors (59).

### Sirolimus (rapamycin).

The mTOR inhibitor sirolimus is increasingly used to treat slow-flow vascular anomalies, such as venous malformations and complex lymphatic malformations (61–63). Based on its effects on HemSCs, sirolimus may be an adjunct or alternative therapy for complex and endangering IH. In vitro studies have shown that sirolimus reduces clonal expansion, stimulates mesenchymal differentiation of HemSCs, and inhibits de novo vessel formation by HemSCs (64). Moreover, sirolimus induced regression of IH blood vessels, consistent with its antiangiogenic activity. In contrast to corticosteroids, sirolimus has no effect on VEGF-A levels in HemSCs (64). This prompted a preclinical combination therapy experiment. Reduced doses of dexamethasone and sirolimus that were ineffective when used singly showed significant inhibition of HemSC vessel formation in mice when combined (64). This study underscores the potential for combining drugs with distinct mechanisms to block vascular overgrowth. Several case reports have described the efficacy of sirolimus alone or in combination with propranolol for IH (65–68). Safety was not a concern in this young patient cohort; sirolimus had been given previously to neonates with extensive lymphatic malformations (69, 70). A prospective randomized trial will be necessary to assess the efficacy and safety of sirolimus for treatment of IH.

### Propranolol.

Propranolol was serendipitously discovered to be an effective treatment for IH in a landmark study by Léauté-Labrèze and colleagues. Two infants were given propranolol for cardiac indications and showed significant regression of a concomitant complicated IH (71). A randomized controlled clinical trial followed (*n* = 460) demonstrating 60% complete or nearly complete IH resolution after propranolol treatment with 3 mg/kg/d. IH usually resolved within 6 months of age when administered early during the proliferative phase; IH regrowth after discontinuation of propranolol occurred in 10% (72). Propranolol is currently the only FDA-approved drug for IH.

Despite its success, propranolol can cause adverse events in infants: hypotension, bradycardia, peripheral vasospasm, diarrhea, hypoglycemia and seizures, bronchospasm, growth retardation, agitation, and sleep disturbance (72–74). As a lipophilic molecule, propranolol crosses the blood-brain barrier and has been reported to impact gross motor skills, such as walking (75). A long-term study on neurocognitive functioning of children aged ≥6 years revealed that male children who had been treated with propranolol or atenolol for IH had significantly lower IQ scores compared with treated female and male children of the general population (76). The potential for concerning untoward effects of propranolol underscores the need for understanding its molecular targets. Knowledge of such targets may help to refine IH therapy to provide maximal effect and minimal adverse events.

Propranolol is a lipophilic, nonselective antagonist of the GPCR β1-and β2-adrenergic receptors that has revolutionized treatment of cardiovascular disease. Its mechanism of action in IH is controversial. A possible inhibitory effect on vascular growth linked to β-adrenergic receptor antagonism has been suggested based on detection of β-adrenergic receptors in IH. Furthermore, decreased cAMP levels and MAPK pathway activation has been shown in propranolol-treated HemSCs (77, 78). Other proposed mechanisms include promotion of vasoconstriction, apoptosis, and inhibition of angiogenic sprouting, nitric oxide production, and an effect on the renin-angiotensin system (79–82). Many of these in vitro studies lack confirmation in vivo, and, moreover, drug concentrations often exceeded the correlative plasma levels found in patients.

Propranolol is a chiral drug that consists of mirror-image, nonsuperimposable molecules in an equimolar (1:1) mix of *S*(–) and *R*(+) enantiomers. The *S*(–) enantiomer of propranolol is a potent antagonist of β1- and β2-adrenergic receptors. The *R*(+) enantiomer is largely devoid of beta blocker activity unless used at high concentrations (83, 84). We decided to test effects of each enantiomer separately on IH-derived cells. *R*(+) propranolol blocked HemSC endothelial differentiation and HemSC de novo vessel formation in mice (85, 86). This suggested the beta blocker activity of propranolol is not required for inhibition of IH and pointed to an *off-target* mechanism of action. It has been reported that enantiomer induced changes in gene expression in HemSCs and in a murine endothelioma cell line, bEnd.3 (87).

### Propranolol and SOX18.

A case report of a patient with a rare vascular disease and unexpectedly minor symptoms, who was treated successfully with propranolol for aortic dilation (88), prompted Francois and colleagues to investigate a possible connection between the transcription factor sex-determining region Y (SRY) box transcription factor 18 (SOX18) and propranolol. The diagnosis was hypotrichosis-lymphedema-telangiectasia and renal syndrome (HLTRS), a disorder caused by a premature stop codon in SOX18 (89), which creates a dominant negative form of SOX18. Studies in the *Ragged Opossum* (*RaOp*) mouse, which has an analogous SOX18 mutation (90) and, thereby, serves as a preclinical model of HLTRS, showed that *R*(+) propranolol was sufficient to inhibit the abnormally extensive corneal neovascularization in the *RaOp* mouse (85). This discovery implicated SOX18 in the mechanism of action for propranolol in HLTRS — independent of β-adrenergic receptors.

SOX18 is a master transcriptional regulator of vascular development and differentiation. It is expressed in nascent blood and lymphatic endothelium as well as in adult endothelial progenitor cells in adults (91). SOX18 plays fundamental roles in arterial specification (92), lymphangiogenesis (93), and tumor angiogenesis (94). SOX18 regulates endothelial gene transcription in a homodimer or heterodimer configuration (95). The proposed mechanism in HLTRS is that mutant SOX18 “poisons” normal SOX18 dimers, resulting in faulty transcriptional complexes (96). Disruption of such poisonous dimers with *R*(+) propranolol in *RaOP* mice was postulated to restore normal SOX18 function (85). Of interest, the Notch signaling regulator recombination signal binding protein for the immunoglobulin k J region (RBPJ) is an important SOX18 protein binding partner (97). The small-molecule inhibitor Sm4 disrupts the transcriptional activity of SOX18 homodimers and SOX18-RBPJ heterodimers by perturbing the protein-protein interaction. This inhibition provides a pharmacologic tool for probing SOX18 function in vivo and in vitro (98). Sm4 inhibition of SOX18 suppresses vascular development in zebrafish, halts both tumor angio- and lymphangiogenesis in a breast cancer model (97, 98), and diminishes endothelial-specific viral responses (99, 100).

**Figure 2:**
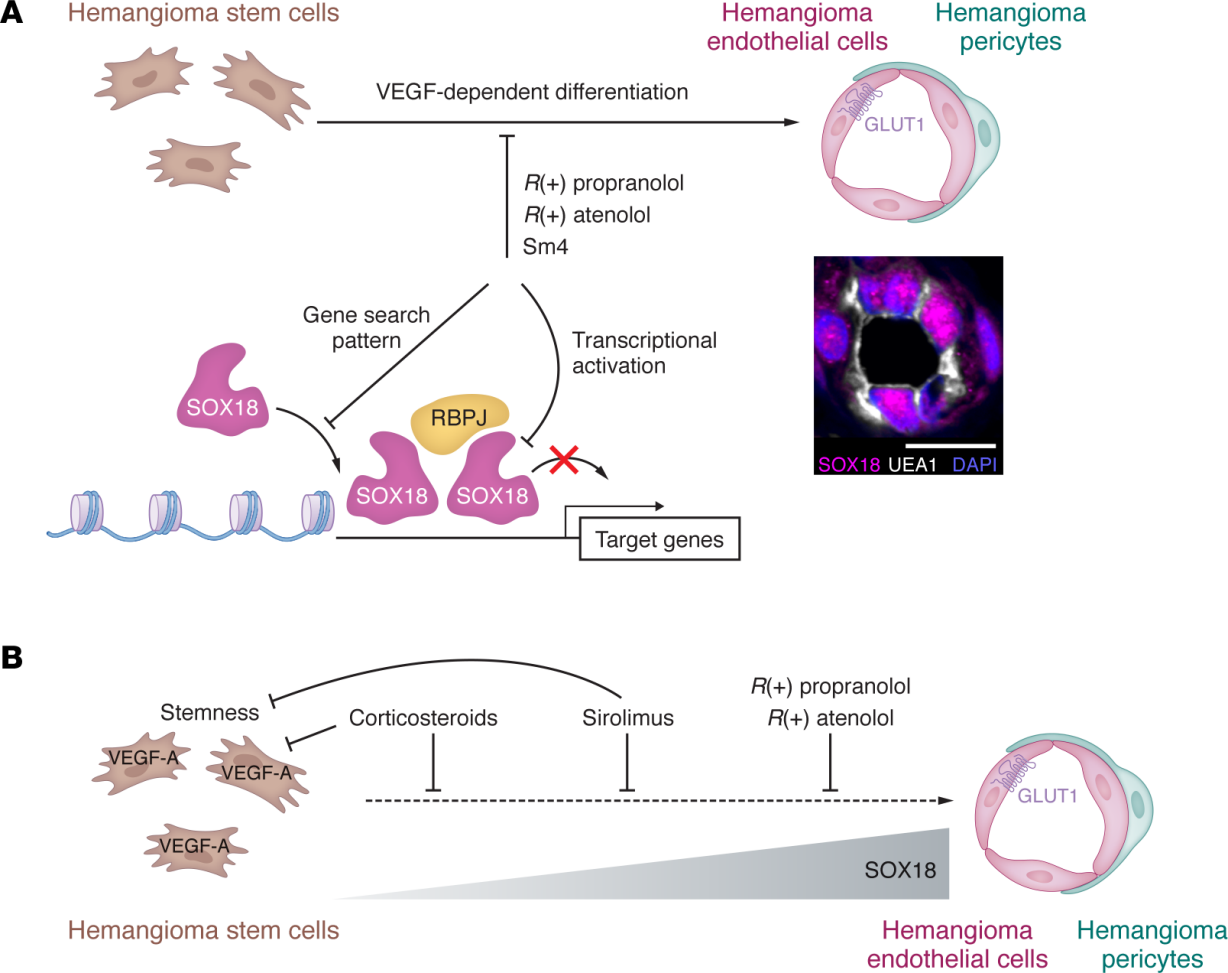
Propranolol targets the endothelial transcription factor SOX18 to inhibit vasculogenesis in IH. (**A**) The *R*(+) enantiomers of propranolol and atenolol and the small-molecule SOX18 inhibitor Sm4 inhibit hemangioma stem cell (HemSC) to hemangioma endothelial cell (HemEC) differentiation in vitro and ability of HemSCs to form de novo vessels in vivo. *R*(+) propranolol inhibits SOX18 by interfering with its search patterns along chromatin, its homodimer (SOX18:SOX18) or heterodimer formation with RBPJ (SOX18:RBPJ), and its transcriptional activation of target genes. In patient tissue, SOX18 expression (magenta) coincides with nuclei (blue) and colocalizes with UEA (gray), indicating its presence in endothelial cells of proliferating IH tissue. The inset confocal image was acquired with a Zeiss LSM 880 by AH. Scale bar: 10 μm. Adapted with permission from the *Journal of Clinical Investigation* (86). (**B**) Summary of differential drug mechanisms of action inhibiting IH vasculogenesis. Corticosteroids inhibit the expression of VEGF-A; sirolimus reduces stemness and self-renewal of HemSC; *R*(+) propranolol and *R*(+) atenolol act on SOX18 as described in **A**. SOX18 expression increases over the course of HemSC to HemEC differentiation.

Studies demonstrating *R*(+) propranolol inhibition of corneal neovascularization in the *RaOp* mouse and the central role of SOX18 in endothelial differentiation and vascular development prompted us to investigate SOX18 and *R*(+) propranolol in IH. Our hypothesis was that SOX18 function in IH is dysregulated, leading to transient vascular overgrowth, i.e., the proliferating phase of IH. *R*(+) propranolol was shown to block HemSC endothelial differentiation at 5 μM as effectively as racemic propranolol or Sm4 (85). In vivo, *R*(+) propranolol inhibited HemSC de novo vessel formation in the xenograft model, comparable to racemic propranolol or Sm4 (86). Real-time single-molecule imaging of SOX18 interaction with chromatin showed that *R*(+) propranolol impeded the ability of SOX18 to survey chromatin, demonstrating on-target drug engagement in live cells (Figure 2). Furthermore, *R*(+) propranolol reduced protein-protein interactions with its dimerization partner RBPJ and reduced transcription of SOX18 target genes NOTCH1 and VCAM1. Finally, SOX18 and RBPJ were colocalized in endothelial nuclei of proliferating phase IH specimens (86).

These experiments strongly implicate SOX18 in propranolol therapy for IH. Our findings suggest that using the *R*(+) enantiomer of propranolol could increase its efficacy and safety in treatment of IH. More studies will be required to unravel the SOX18 transcriptional targets that drive the dysregulated vascular growth in IH and potentially in IH involution. In a broader perspective, and based on propranolol use for other vascular anomaly entities (101–103), it is tempting to speculate that SOX18, as a key regulator of angio- and lymphangiogenesis, may also play a role in other vascular anomalies. Selective inhibitors of SOX18 and its transcriptional targets may provide novel therapies for these vascular anomaly entities.

### Other beta blockers.

Alternatives to propranolol, oral atenolol and nadolol, have been used to treat IH. As a selective β1-adrenergic receptor blocker, atenolol has a lower risk of bronchospasm and hypoglycemia (104). Like propranolol, atenolol is a combination of *R*(+) and *S*(–) enantiomers, and the *R*(+) enantiomer is devoid of beta blocker activity (84). Seebauer and colleagues showed that *R*(+) atenolol blocks HemSC endothelial differentiation in vitro and HemSC vessel formation in vivo at the same dosage as *R*(+) propranolol. This suggests that atenolol exerts a β-adrenergic receptor–independent effect in IH, analogous to the *R*(+) enantiomer of propranolol (86).

Nadolol is a nonselective β1- and β2-adrenergic receptor blocker with no intrinsic sympathomimetic activity and little myocardial depressant effect (105). Caution is warranted regarding its pharmacokinetics. Nadolol is not metabolized and is excreted unchanged mostly in feces. Thus, gastrointestinal passage results in reabsorption and accumulation. The drug has been largely abandoned after one reported death of an infant with IH linked to nadolol (106). Furthermore, a murine model demonstrated that beta blockers induce the release of nitric oxide and nitric peroxide in the hypothalamus as a result of their ability to cross the blood-brain barrier and, hence, may have deleterious neurological side effects (107).

As an alternative to systemic beta blockers, a topical timolol maleate solution (0.5%) was evaluated for early treatment of IH in infants younger than 60 days. A randomized phase IIa pilot clinical trial (*n* = 69) demonstrated that, while timolol is well tolerated, it does not significantly improve the outcome (108).

### Alternative drugs and treatments.

Historically, vincristine and α interferon were used, but they are no longer recommended, considering their unfavorable safety and outcomes (18). The ACE inhibitor captopril has been suggested as an alternative to beta blockers based on the proposed implications of the renin-angiotensin system in the pathophysiology of IH. Its efficacy was shown to be inferior to propranolol (109).

Pulse-dye laser therapy for residual telangiectasia and discoloration of involuted IH is well established (18). Evidence is lacking for early laser therapy in proliferating IH to prevent growth or to treat ulcerated IH (110). Interventional procedures in the management of IH have been largely superseded by medical treatment. A crucial role remains for properly timed and executed resection and/or interventional radiology procedures for those IHs that are life-threatening (e.g., causing acute airway obstruction or large tumors resulting in high-output cardiac failure), function-impairing (e.g., vision), and/or are recalcitrant to medical treatment (111, 112).

## Unresolved mysteries in IH

### Genetics of IH.

The possibility of germline mutations and familial occurrence has been proposed (113–115), yet despite great effort in the field, there have been few advances in uncovering what might be genetic variants in the etiology of IH (116). This contrasts with the identification of several somatic and germline mutations in vascular malformations and other vascular tumors over the past 25 years (117). Most IHs occur sporadically without a hereditary component; this is supported by identical twin studies (118). There is a report, however, of monozygotic twins with nearly identical periorbital IH (115). In summary, the lack of a consistent somatic or germline mutation raises the possibility that epigenetic events play a role in the etiology of IH. A better understanding of the molecular drivers in IH, such as the transcription factor SOX18 and its targets, may provide clues in terms of the epigenetic causes of IH as well as advance our understanding of novel therapeutic targets.

### Origin of HemSC.

When and where a normal counterpart to HemSCs might function in neonatal growth and development is a critical question. HemSCs are functionally akin to vascular progenitor cells, which have been long speculated upon and for which experimental evidence has been rigorously debated. Smoller and Apfelberg boldly speculated that the mitotic cells dispersed among IH vessels might be a primitive cell capable of differentiating into endothelial cells and pericytes (119) — i.e., vascular progenitors. VEGFR2^+^ cells arising from mouse embryonic stem cells were shown to differentiate into endothelial and mural cells and to incorporate as such in the developing vasculature in vivo (120); similar vascular progenitors were subsequently derived from human embryonic stem cells (121). More recently, a CD45^–^CD34^+^CD144^+^CD31^lo^ cell population isolated from term human placenta showed mesenchymal and endothelial differentiative capability (122). The presence of such vascular progenitors in placenta is intriguing, given that IH has been suggested to arise from placental endothelial cells (123). Whether HemSCs arise from a specific location, such as the placenta and disseminate to other locations or arise in situ from resident vascular progenitors has not been tested experimentally in part due to lack of transgenic models. Transcriptomic profiling of IH cells may provide insights on the developmental history of HemSC.

### Mechanisms of involution.

While our understanding of the proliferating phase of IH has advanced, less is known about the molecular program driving the spontaneous involution and transformation to a fibrofatty residuum. Three reports showed increased apoptosis and decreased Bcl-2, an antiapoptotic protein, in involuting IH (124–126) but further molecular mechanisms of involution are yet to be discovered, presenting an important knowledge gap. A long-term follow-up study revealed that 72.4% of patients with IH treated with oral propranolol develop residual sequelae (127) such as fibrofatty residual and expanded skin requiring resection. In vitro studies showed that propranolol accelerates the adipogenic differentiation of HemSCs initially and induced cell death later (128, 129). Both studies employed dosages 100–1,000 times higher than current clinical levels and, therefore, may not accurately reflect current treatment. Uncovering the molecular role of propranolol and the differential effects of its enantiomers on the involuting processes may help toward gaining a better understanding of the complex mechanism governing spontaneous involution in IH.

### The rebound phenomenon.

Up to 25% of IHs with initially good response to propranolol regrow after discontinuation — an observation that is referred to as *rebound phenomenon*. Predictive factors for IH regrowth include discontinuation of propranolol treatment earlier than 9 months of age, deep IH component, and emergence in a female child (130). Why a subgroup of IH regrow is not yet fully understood. We speculate that regrowth occurs when remaining HemSCs in the tumor, perhaps in a dormant state, are stimulated by an unknown molecular event to reactivate hemangiogenesis. Alternatively, a second-hit genetic mechanism could occur to restart or incite the vasculogenic processes.

## Conclusions

IH is a remarkable example of vascular overgrowth and regression: blood vessels form rapidly, only to undergo a slow spontaneous involution. Understanding hemangiogenesis may provide insights on fundamental mechanisms of postnatal human vascular development and quiescence; however, many unanswered questions remain. Deciphering the mechanisms of serendipitously discovered drugs in treating IH has provided important clues on the molecular players in IH, e.g., VEGF-A and SOX18. It remains to be determined, however, what type(s) of genetic or epigenetic alterations govern the natural life cycle of IH. If such alterations are identified, we will gain a foothold to address some of the unresolved mysteries and perhaps understand why malignant transformation does not occur and what instead drives spontaneous regression in IH. In parallel with uncovering mechanisms, further investigations are needed to continue to improve safe therapeutic options for IH to alleviate the all-too-often disfigurement and destruction of tissue and organ and potentially life-threatening complications that can occur in some patients with IH.

## Version 1 04/15/2024

Electronic publication

## References

[1] Leaute-Labreze C (2017). Infantile haemangioma. Lancet.

[2] Anderson KR (2016). Increasing incidence of infantile hemangiomas (IH) over the past 35 years: Correlation with decreasing gestational age at birth and birth weight. J Am Acad Dermatol.

[3] Ding Y (2020). Risk factors for infantile hemangioma: a meta-analysis. World J Pediatr.

[4] Couto RA (2012). Infantile hemangioma: clinical assessment of the involuting phase and implications for management. Plast Reconstr Surg.

[5] Bauland CG (2011). Untreated hemangiomas: growth pattern and residual lesions. Plast Reconstr Surg.

[6] Maguiness S (2015). Rapidly involuting congenital hemangioma with fetal involution. Pediatr Dermatol.

[7] North PE (2000). GLUT1: a newly discovered immunohistochemical marker for juvenile hemangiomas. Hum Pathol.

[8] Ayturk UM (2016). Somatic activating mutations in GNAQ and GNA11 are associated with congenital hemangioma. Am J Hum Genet.

[9] Johnson A (2021). Presence of estrogen and progesterone receptors in proliferating and involuting infantile hemangiomas. J Plast Reconstr Aesthet Surg.

[10] Xiao X (2004). Synergistic effect of estrogen and VEGF on the proliferation of hemangioma vascular endothelial cells. J Pediatr Surg.

[11] Zhang L (2017). Estrogen-mediated hemangioma-derived stem cells through estrogen receptor-α for infantile hemangioma. Cancer Manag Res.

[12] Chiller KG (2002). Hemangiomas of infancy: clinical characteristics, morphologic subtypes, and their relationship to race, ethnicity, and sex. Arch Dermatol.

[13] Wassef M (2015). Vascular anomalies classification: recommendations from the international society for the study of vascular anomalies. Pediatrics.

[14] Haggstrom AN (2006). Patterns of infantile hemangiomas: new clues to hemangioma pathogenesis and embryonic facial development. Pediatrics.

[15] Reimer A (2016). Anatomical patterns of infantile hemangioma (IH) of the extremities (IHE). J Am Acad Dermatol.

[16] Haggstrom AN (2010). Risk for PHACE syndrome in infants with large facial hemangiomas. Pediatrics.

[17] Forde KM (2017). Segmental hemangioma of the head and neck: High prevalence of PHACE syndrome. J Am Acad Dermatol.

[18] Krowchuk DP (2019). Clinical practice guideline for the management of infantile hemangiomas. Pediatrics.

[19] Iacobas I (2010). LUMBAR: association between cutaneous infantile hemangiomas of the lower body and regional congenital anomalies. J Pediatr.

[20] Stockman A (2007). SACRAL syndrome: spinal dysraphism, anogenital, cutaneous, renal and urologic anomalies, associated with an angioma of lumbosacral localization. Dermatology.

[21] Suh KY, Frieden IJ (2010). Infantile hemangiomas with minimal or arrested growth: a retrospective case series. Arch Dermatol.

[22] Ma EH (2017). Infantile hemangioma with minimal or arrested growth: further observations on clinical and histopathologic findings of this unique but underrecognized entity. Pediatr Dermatol.

[23] Mahon C (2021). Routine liver ultrasound screening does not alter clinical management in a cohort study of multiple cutaneous infantile haemangioma. Br J Dermatol.

[24] Metry DW (2004). Association of solitary, segmental hemangiomas of the skin with visceral hemangiomatosis. Arch Dermatol.

[25] Gong X (2022). Infantile hepatic hemangiomas: looking backwards and forwards. Precis Clin Med.

[26] Chamlin SL (2007). Multicenter prospective study of ulcerated hemangiomas. J Pediatr.

[27] Zhao J (2019). Periocular infantile hemangiomas: Characteristics, ocular sequelae, and outcomes. Pediatr Dermatol.

[28] Czechowicz JA (2021). Airway hemangiomas in PHACE syndrome: a multicenter experience. Otolaryngol Head Neck Surg.

[29] Huang SA (2000). Severe hypothyroidism caused by type 3 iodothyronine deiodinase in infantile hemangiomas. N Engl J Med.

[31] Huang L (2015). Glucose transporter 1-positive endothelial cells in infantile hemangioma exhibit features of facultative stem cells. Stem Cells.

[32] Khan ZA (2008). Multipotential stem cells recapitulate human infantile hemangioma in immunodeficient mice. J Clin Invest.

[33] Xu D (2011). Isolation, characterization, and in vitro propagation of infantile hemangioma stem cells and an in vivo mouse model. J Hematol Oncol.

[34] Mai HM (2013). CD133 selected stem cells from proliferating infantile hemangioma and establishment of an in vivo mice model of hemangioma. Chin Med J (Engl).

[35] Harbi S (2016). Infantile hemangioma originates from a dysregulated but not fully transformed multipotent stem cell. Sci Rep.

[36] Edwards AK (2017). NOTCH3 regulates stem-to-mural cell differentiation in infantile hemangioma. JCI Insight.

[37] Hu W (2021). NOGOB receptor-mediated RAS signaling pathway is a target for suppressing proliferating hemangioma. JCI Insight.

[38] Boye E (2001). Clonality and altered behavior of endothelial cells from hemangiomas. J Clin Invest.

[39] Khan ZA (2006). Endothelial progenitor cells from infantile hemangioma and umbilical cord blood display unique cellular responses to endostatin. Blood.

[40] Jinnin M (2008). Suppressed NFAT-dependent VEGFR1 expression and constitutive VEGFR2 signaling in infantile hemangioma. Nat Med.

[41] Picard A (2008). IGF-2 and FLT-1/VEGF-R1 mRNA levels reveal distinctions and similarities between congenital and common infantile hemangioma. Pediatr Res.

[42] Lee D (2014). Propranolol targets contractility of infantile hemangioma-derived pericytes. B J Dernatol.

[43] Dosanjh A (2000). In vitro characteristics of neonatal hemangioma endothelial cells: similarities and differences between normal neonatal and fetal endothelial cells. J Cutan Pathol.

[44] Boscolo E (2013). Pericytes from infantile hemangioma display proangiogenic properties and dysregulated angiopoietin-1. Arterioscler Thromb Vasc Biol.

[45] Wang FQ (2013). M2-polarised macrophages in infantile haemangiomas: correlation with promoted angiogenesis. J Clin Pathol.

[46] Zhang W (2015). Macrophages contribute to the progression of infantile hemangioma by regulating the proliferation and differentiation of hemangioma stem cells. J Invest Dermatol.

[47] Moisan F (2021). Critical role of Aquaporin-1 and telocytes in infantile hemangioma response to propranolol beta blockade. Proc Natl Acad Sci U S A.

[48] Drolet BA, Frieden IJ (2010). Characteristics of infantile hemangiomas as clues to pathogenesis: does hypoxia connect the dots?. Arch Dermatol.

[49] Kleinman ME (2007). Hypoxia-induced mediators of stem/progenitor cell trafficking are increased in children with hemangioma. Arterioscler Thromb Vasc Biol.

[50] Gomez-Acevedo H (2020). Identification of putative biomarkers for Infantile Hemangiomas and Propranolol treatment via data integration. Sci Rep.

[51] Greenberger S (2010). Corticosteroid suppression of VEGF-A in infantile hemangioma-derived stem cells. N Engl J Med.

[52] Boscolo E (2011). VEGFR-1 mediates endothelial differentiation and formation of blood vessels in a murine model of infantile hemangioma. Am J Pathol.

[53] Zhao B (2017). The Nogo-B receptor promotes Ras plasma membrane localization and activation. Oncogene.

[54] Boscolo E (2011). JAGGED1 signaling regulates hemangioma stem cell-to-pericyte/vascular smooth muscle cell differentiation. Arterioscler Thromb Vasc Biol.

[55] Itinteang T (2014). Biology of infantile hemangioma. Front Surg.

[56] Strub GM (2016). Endothelial and circulating C19MC microRNAs are biomarkers of infantile hemangioma. JCI Insight.

[57] Mong EF (2018). Modulation of LIN28B/Let-7 Signaling by Propranolol Contributes to Infantile Hemangioma Involution. Arterioscler Thromb Vasc Biol.

[58] Katz HP, Askin J (1968). Multiple hemangiomata with thrombopenia. An unusual case with comments on steroid therapy. Am J Dis Child.

[59] Aly MM (2015). Therapeutic superiority of combined propranolol with short steroids course over propranolol monotherapy in infantile hemangioma. Eur J Pediatr.

[60] Pope E (2007). Oral versus high-dose pulse corticosteroids for problematic infantile hemangiomas: a randomized, controlled trial. Pediatrics.

[61] Hammer J (2018). Sirolimus is efficacious in treatment for extensive and/or complex slow-flow vascular malformations: a monocentric prospective phase II study. Orphanet J Rare Dis.

[62] Van Damme A (2020). New and emerging targeted therapies for vascular malformations. Am J Clin Dermatol.

[63] Seront E (2023). Preliminary results of the European multicentric phase III trial regarding sirolimus in slow-flow vascular malformations. JCI Insight.

[64] Greenberger S (2011). Rapamycin suppresses self-renewal and vasculogenic potential of stem cells isolated from infantile hemangioma. J Invest Dermatol.

[65] Kaylani S (2013). Treatment of infantile hemangiomas with sirolimus in a patient with PHACE syndrome. Pediatr Dermatol.

[66] Hutchins KK (2017). Treatment of refractory infantile hemangiomas and pulmonary hypertension with sirolimus in a pediatric patient. J Pediatr Hematol Oncol.

[67] Davila-Osorio VL (2020). Propranolol-resistant infantile hemangioma successfully treated with sirolimus. Pediatr Dermatol.

[68] Kleinman EP (2023). Sirolimus for diffuse intestinal infantile hemangioma with PHACE features: systematic review. Pediatr Res.

[69] Holm A (2021). Efficacy of sirolimus in patients requiring tracheostomy for life-threatening lymphatic malformation of the head and neck: a report from the European reference network. Front Pediatr.

[70] Triana P (2019). Oral sirolimus: an option in the management of neonates with life-threatening upper airway lymphatic malformations. Lymphat Res Biol.

[71] Leaute-Labreze C (2008). Propranolol for severe hemangiomas of infancy. N Engl J Med.

[72] Leaute-Labreze C (2015). A randomized, controlled trial of oral propranolol in infantile hemangioma. N Engl J Med.

[73] Leaute-Labreze C (2016). Safety of oral propranolol for the treatment of infantile hemangioma: a systematic review. Pediatrics.

[74] Pope E (2021). Commentary:Beta-blockers and sleep problems. Pediatr Dermatol.

[75] Gonski K, Wargon O (2014). Retrospective follow up of gross motor development in children using propranolol for treatment of infantile haemangioma at Sydney Children’s Hospital. Australas J Dermatol.

[76] Hermans MM (2023). Long-term neurocognitive functioning of children treated with propranolol or atenolol for infantile hemangioma. Eur J Pediatr.

[77] Munabi NC (2016). Propranolol targets hemangioma stem cells via cAMP and mitogen-activated protein kinase regulation. Stem Cells Transl Med.

[78] Lee JC (2021). Propranolol therapy in infantile hemangioma: it is not just about the beta. Plast Reconstr Surg.

[79] Stiles J (2012). Propranolol treatment of infantile hemangioma endothelial cells: A molecular analysis. Exp Ther Med.

[80] Wong A (2012). Propranolol accelerates adipogenesis in hemangioma stem cells and causes apoptosis of hemangioma endothelial cells. Plast Reconstr Surg.

[81] Wnek A (2017). Molecular and immunohistochemical expression of apoptotic proteins Bax, Bcl-2 and Caspase 3 in infantile hemangioma tissues as an effect of propranolol treatment. Immunol Lett.

[82] Sharifpanah F (2014). β-Adrenergic receptor antagonists inhibit vasculogenesis of embryonic stem cells by downregulation of nitric oxide generation and interference with VEGF signalling. Cell Tissue Res.

[83] Mehvar R, Brocks DR (2001). Stereospecific pharmacokinetics and pharmacodynamics of beta-adrenergic blockers in humans. J Pharm Pharm Sci.

[84] Stoschitzky K (1995). Stereoselective vascular effects of the (R)- and (S)-enantiomers of propranolol and atenolol. J Cardiovasc Pharmacol.

[85] Overman J (2019). R-propranolol is a small molecule inhibitor of the SOX18 transcription factor in a rare vascular syndrome and hemangioma. Elife.

[86] Seebauer CT (2022). Non-beta blocker enantiomers of propranolol and atenolol inhibit vasculogenesis in infantile hemangioma. J Clin Invest.

[87] Sasaki M (2019). Propranolol exhibits activity against hemangiomas independent of beta blockade. NPJ Precis Oncol.

[88] Wunnemann F (2016). Aortic dilatation associated with a de novo mutation in the SOX18 gene: expanding the clinical spectrum of hypotrichosis-lymphedema-telangiectasia syndrome. Can J Cardiol.

[89] Irrthum A (2003). Mutations in the transcription factor gene SOX18 underlie recessive and dominant forms of hypotrichosis-lymphedema-telangiectasia. Am J Hum Genet.

[90] Pennisi D (2000). Mutations in Sox18 underlie cardiovascular and hair follicle defects in ragged mice. Nat Genet.

[91] Patel J (2017). Functional definition of progenitors versus mature endothelial cells reveals key SoxF-dependent differentiation process. Circulation.

[92] Herpers R (2008). Redundant roles for sox7 and sox18 in arteriovenous specification in zebrafish. Circ Res.

[93] Francois M (2008). Sox18 induces development of the lymphatic vasculature in mice. Nature.

[94] Young N (2006). Effect of disrupted SOX18 transcription factor function on tumor growth, vascularization, and endothelial development. J Natl Cancer Inst.

[95] Moustaqil M (2018). Homodimerization regulates an endothelial specific signature of the SOX18 transcription factor. Nucleic Acids Res.

[96] McCann AJ (2021). A dominant-negative SOX18 mutant disrupts multiple regulatory layers essential to transcription factor activity. Nucleic Acids Res.

[97] Overman J (2017). Pharmacological targeting of the transcription factor SOX18 delays breast cancer in mice. Elife.

[98] Fontaine F (2017). Small-molecule inhibitors of the SOX18 transcription factor. Cell Chem Biol.

[99] Gramolelli S (2020). Oncogenic herpesvirus engages endothelial transcription factors SOX18 and PROX1 to increase viral genome copies and virus production. Cancer Res.

[100] Tuohinto K (2023). KSHV infection of endothelial precursor cells with lymphatic characteristics as a novel model for translational Kaposi’s sarcoma studies. PLoS Pathog.

[101] Lanfranconi S (2023). Safety and efficacy of propranolol for treatment of familial cerebral cavernous malformations (Treat_CCM): a randomised, open-label, blinded-endpoint, phase 2 pilot trial. Lancet Neurol.

[102] Albarki H, Rimmer J (2022). The use of beta-blockers in hereditary hemorrhagic telangiectasia-related epistaxis: a systematic review. Am J Rhinol Allergy.

[103] Wu JK (2016). Initial experience with propranolol treatment of lymphatic anomalies: a case series. Pediatrics.

[104] Bayart CB (2017). Atenolol versus propranolol for treatment of infantile hemangiomas during the proliferative phase: a retrospective noninferiority study. Pediatr Dermatol.

[105] Bernabeu-Wittel J (2015). Oral nadolol for children with infantile hemangiomas and sleep disturbances with oral propranolol. Pediatr Dermatol.

[106] McGillis E (2020). Death associated with nadolol for infantile hemangioma: a case for improving safety. Pediatrics.

[107] Laurens C (2019). Central effects of beta-blockers may be due to nitric oxide and hydrogen peroxide release independently of their ability to cross the blood-brain barrier. Front Neurosci.

[108] Munoz-Garza FZ (2021). Efficacy and safety of topical timolol for the treatment of infantile hemangioma in the early proliferative stage: A Randomized Clinical Trial. JAMA Dermatol.

[109] Zaher H (2016). Propranolol versus captopril in the treatment of infantile hemangioma (IH): A randomized controlled trial. J Am Acad Dermatol.

[110] Kwon SH (2014). Effect of early long-pulse pulsed dye laser treatment in infantile hemangiomas. Dermatol Surg.

[111] Cheng J (2019). Outcomes of surgical treatment for hemangiomas. Pediatr Dermatol.

[112] Lord DJ, Chennapragada SM (2011). Embolization in neonates and infants. Tech Vasc Interv Radiol.

[113] Blei F (1998). Familial segregation of hemangiomas and vascular malformations as an autosomal dominant trait. Arch Dermatol.

[114] Walter JW (1999). Genetic mapping of a novel familial form of infantile hemangioma. Am J Med Genet.

[115] Castren E (2016). Inheritance patterns of infantile hemangioma. Pediatrics.

[116] Cheraghlou S (2019). Genetic investigation of childhood vascular tumor biology reveals pathways for therapeutic intervention. F1000Res.

[117] Queisser A (2021). Genetic basis and therapies for vascular anomalies. Circ Res.

[118] Cheung DS (1997). Hemangioma in twins. Ann Plast Surg.

[119] Smoller BR, Apfelberg DB (1993). Infantile (juvenile) capillary hemangioma: a tumor of heterogeneous cellular elements. J Cutan Pathol.

[120] Yamashita J (2000). Flk1-positive cells derived from embryonic stem cells serve as vascular progenitors. Nature.

[121] Sone M (2007). Pathway for differentiation of human embryonic stem cells to vascular cell components and their potential for vascular regeneration. Arterioscler Thromb Vasc Biol.

[122] Shafiee A (2018). Meso-endothelial bipotent progenitors from human placenta display distinct molecular and cellular identity. Stem Cell Reports.

[123] Barnes CM (2005). Evidence by molecular profiling for a placental origin of infantile hemangioma. Proc Natl Acad Sci U S A.

[124] Razon MJ (1998). Increased apoptosis coincides with onset of involution in infantile hemangioma. Microcirculation.

[125] Iwata J (1996). High frequency of apoptosis in infantile capillary haemangioma. J Pathol.

[126] Mancini AJ, Smoller BR (1996). Proliferation and apoptosis within juvenile capillary hemangiomas. Am J Dermatopathol.

[127] Yu Z (2019). Clinical and radiological outcomes of infantile hemangioma treated with oral propranolol: A long-term follow-up study. J Dermatol.

[128] England RW (2014). Propranolol promotes accelerated and dysregulated adipogenesis in hemangioma stem cells. Ann Plast Surg.

[129] Li HH (2019). Propranolol accelerats hemangioma stem cell transformation into adipocyte. Ann Plast Surg.

[130] Shah SD (2016). Rebound growth of infantile hemangiomas after propranolol therapy. Pediatrics.

[132] Boscolo E, Bischoff J (2009). Vasculogenesis in infantile hemangioma. Angiogenesis.

[133] Greenberger S, Bischoff J (2013). Pathogenesis of infantile haemangioma. Br J Dermatol.

